# Biocompatible Short-Peptides Fibrin Co-assembled Hydrogels

**DOI:** 10.1021/acsapm.2c02164

**Published:** 2023-02-21

**Authors:** Cristina Gila-Vilchez, Mari Carmen Mañas-Torres, Óscar Darío García-García, Alfredo Escribano-Huesca, Laura Rodríguez-Arco, Víctor Carriel, Ismael Rodriguez, Miguel Alaminos, Modesto Torcuato Lopez-Lopez, Luis Álvarez de Cienfuegos

**Affiliations:** † Departamento de Física Aplicada, Universidad de Granada (UGR), C. U. Fuentenueva, Avenida Severo Ochoa s/n, E-18071 Granada, Spain; ‡ Departamento de Química Orgánica, Unidad de Excelencia Química Aplicada a Biomedicina y Medioambiente (UEQ), Universidad de Granada (UGR), C. U. Fuentenueva, Avenida Severo Ochoa s/n, E-18071 Granada, Spain; § Department of Histology, Universidad de Granada (UGR), Avenida de Madrid 11, 18012 Granada, Spain; ∥ Instituto de Investigación Biosanitaria ibs.GRANADA, Avenida de Madrid, 15, 18016, Granada, Spain

**Keywords:** peptides, self-assembly, supramolecular hydrogels, composite hydrogels, tissue engineering, regenerative medicine

## Abstract

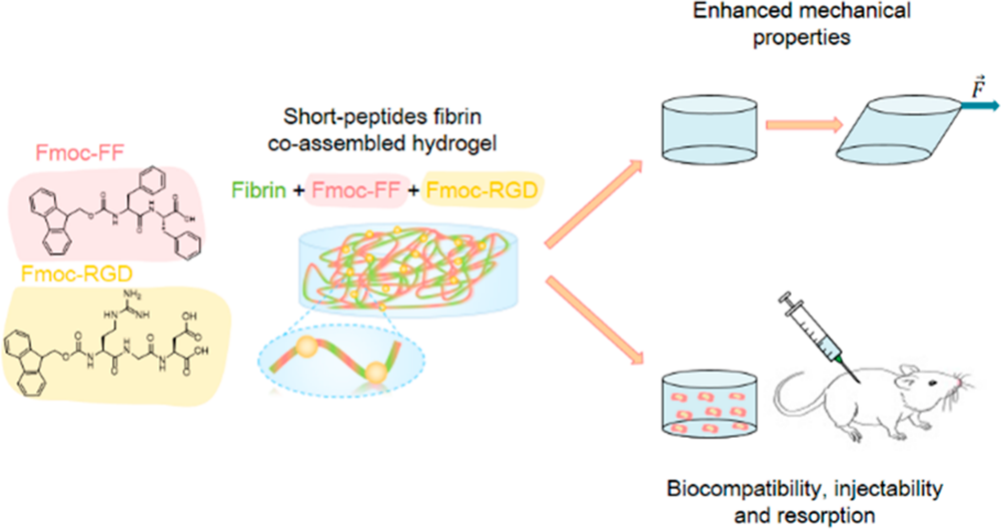

Fibrin hydrogels made by self-assembly of fibrinogen obtained from human plasma have shown excellent biocompatible and biodegradable properties and are widely used in regenerative medicine. The fibrinogen self-assembly process can be triggered under physiological conditions by the action of thrombin, allowing the injection of pregel mixtures that have been used as cell carriers, wound-healing systems, and bio-adhesives. However, access to fibrinogen from human plasma is expensive and fibrin gels have limited mechanical properties, which make them unsuitable for certain applications. One solution to these problems is to obtain composite gels made of fibrin and other polymeric compounds that improve their mechanical properties and usage. Herein, we prepared composite hydrogels made by the self-assembly of fibrinogen together with Fmoc-FF (Fmoc-diphenylalanine) and Fmoc-RGD (Fmoc-arginine-glycine-aspartic acid). We have shown that the mixture of these three peptides co-assembles and gives rise to a unique type of supramolecular fiber, whose morphology and mechanical properties can be modulated. We have carried out a complete characterization of these materials from chemical, physical, and biological points of view. Composite gels have improved mechanical properties compared to pure fibrin gels, as well as showing excellent biocompatibility ex vivo. In vivo experiments have shown that these gels do not cause any type of inflammatory response or tissue damage and are completely resorbed in short time, which would enable their use as vehicles for cell, drug, or growth factor release.

## Introduction

Short-peptide supramolecular hydrogels are extremely versatile materials with remarkable bio- and technological applications.^1−3^ Representative examples of this family are tri- and dipeptides containing an aromatic fluorenylmethoxicarbonyl (Fmoc) or naphthyl (Nap) protecting group.^1,4^ From a synthetic point of view, these molecules are very simple, which makes them easily accessible and economically affordable, and many of them are commercially available. All of these factors have contributed to broadening the use of these peptides to develop and explore materials.^5−10^ Another key characteristic of these peptides is their ability to self-assemble under the application of different stimuli, such as solvent or pH switch, addition of salts, or enzymatic reactions.^1,4^ This can be exploited to induce gelation under physiological conditions, making them highly compatible with biomedical applications.^11^ Furthermore, their resulting macroscopic and physical properties can also be affected or tuned by different stimuli offering an extra degree of versatility.^12,13^ In addition, control over the stimulus-induced transition is also crucial to obtain composite or hybrid hydrogels made by the combination of several organic compounds^14,15^ or mixtures of organic and inorganic/metallic substrates.^5,16−21^ At such, Adams et al. have shown that mixtures of different Nap-dipeptides, under specific pH conditions, can form copolymers (co-assembly) or individual homopolymers (self-sorting) based on the relative p*K*_a_ of the amino acids involved.^22−25^ We have recently shown that Fmoc-FF (Fmoc-diphenylalanine) is able to promote the co-assembly of different Fmoc- and Nap-dipeptides giving rise to hydrogels having different mechanical properties.^26^ The mechanism of growth of these peptides usually follows a nucleation–elongation mechanism in which, starting from an initial metastable phase, the formation of fibers is triggered when the conditions allow overpassing the free energy barrier of polymerization.^27−29^ We have shown that metastable intermediates formed by the combination of two different peptides had a lower free energy barrier of polymerization; therefore, the co-assembly was favored vs the self-assembly of individual peptides.^26^ Hydrogels in which the polymeric network is made by the co-assembly of different peptides have many advantages,^30,31^ in particular, for tissue engineering.^32−35^ For example, the peptide network can contain bioactive molecules that can promote cell adhesion, as it has been well-studied by Ulijn et al., using the combination of Fmoc-FF and Fmoc-RGD (Fmoc-arginine-glycine-aspartic acid), containing the bioactive RGD residue shown to promote cell adhesion.^33^ The density of the bioactive molecules can be easily controlled, and its incorporation in the network warranties its homogeneous distribution. Moreover, since the combination of the two peptides affords one type of polymeric network, the hydrogel porosity and the diffusion of substrates through it are less affected, being that these two factors are essential for cell growth and proliferation. This cannot be attained when short-peptide hydrogels are mixed with polymeric or other kinds of compounds in which only composite materials with different micro- and macroscopic properties can be obtained. Remarkably, this later strategy has been implemented successfully to develop composite hydrogels for biomedical applications. As such, composite hydrogels made of Fmoc-FF and hyaluronic acid have been shown to improve the mechanical properties of hyaluronic acid gels alone, being useful for drug delivery applications.^36^ Fmoc-FF has also been used in combination with alginate polymers. This combination has been tested for different biomedical applications including cell culture and drug delivery.^37−39^ Nap-FF derivatives have been mixed with silk fibroin to afford injectable hydrogels for tissue engineering.^40,41^ Nap-FF not only improved the mechanical properties of the resulting gels, but it was also able to trigger the conformational transition of silk fibroin from random coil to β-sheet.^40^ Recently, we have been able to obtain hybrid injectable hydrogels by mixing Fmoc-FF with magnetic nanoparticles.^42^ In this case, the incorporation of magnetic nanoparticles significantly improved the mechanical properties of the hydrogels and, at the same time, allowed control of these properties remotely by external magnetic fields.

**Figure 1:**
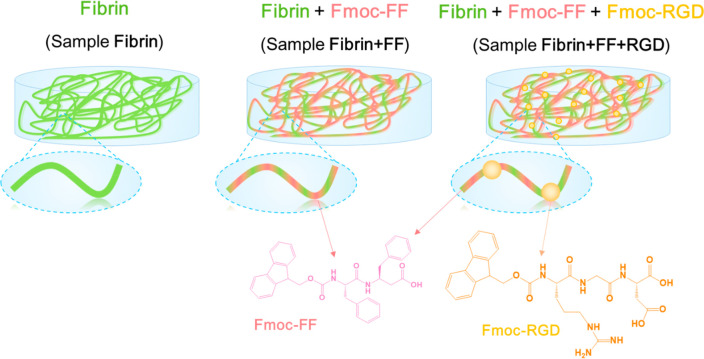
Schematic drawing of the co-assembled peptide hydrogels for regenerative medicine developed in this work.

Considering this, herein we have designed supramolecular hydrogels made by the co-assembly of Fmoc-FF and/or Fmoc-RGD and fibrin (Figure 1). Specifically, supramolecular hydrogels have been formed by the combination of different ratios of Fmoc-FF, Fmoc-RGD peptide solutions with fibrin precursors (fibrinogen) obtained from human plasma. Fibrin hydrogels are very useful biomaterials for regenerative medicine since they promote cell attachment and present excellent biocompatibility and biodegradability.^43^ The formation of these hydrogels can be triggered in situ, and this property has been exploited to develop injectable carriers to repair damaged tissues.^44^ Fibrin hydrogels have been used as bio-adhesives for wound closure in surgeries and as cell carriers.^45^ These gels show minimal inflammation reaction, and their degradation rate can be controlled to match tissue regeneration.^46^ For some applications, the mechanical properties of fibrin gels are not adequate, making them fragile and difficult to handle. To avoid this, fibrin gels have been combined with different natural or synthetic materials, giving rise to composite hydrogels with improved mechanical properties.^47−51^ Nevertheless, and as far as we know, the combination of short-peptide supramolecular hydrogels with fibrin has not been studied yet.

Our results show that the three peptides (Fmoc-FF, Fmoc-RGD, and fibrinogen) are able to co-assemble, giving rise to supramolecular fibers of well-defined morphology. The resulting composite hydrogels show improved mechanical and macroscopic properties, as well as excellent biocompatibility and biodegradability ex vivo and in vivo. The possibility to modulate the properties of fibrin hydrogels by simply mixing them with small proportions of synthetic short peptides paves the way to develop more affordable biocompatible materials with a myriad of potential biomedical applications.

## Results and Discussion

### Physicochemical Characterization of the Hydrogels

**Figure 2:**
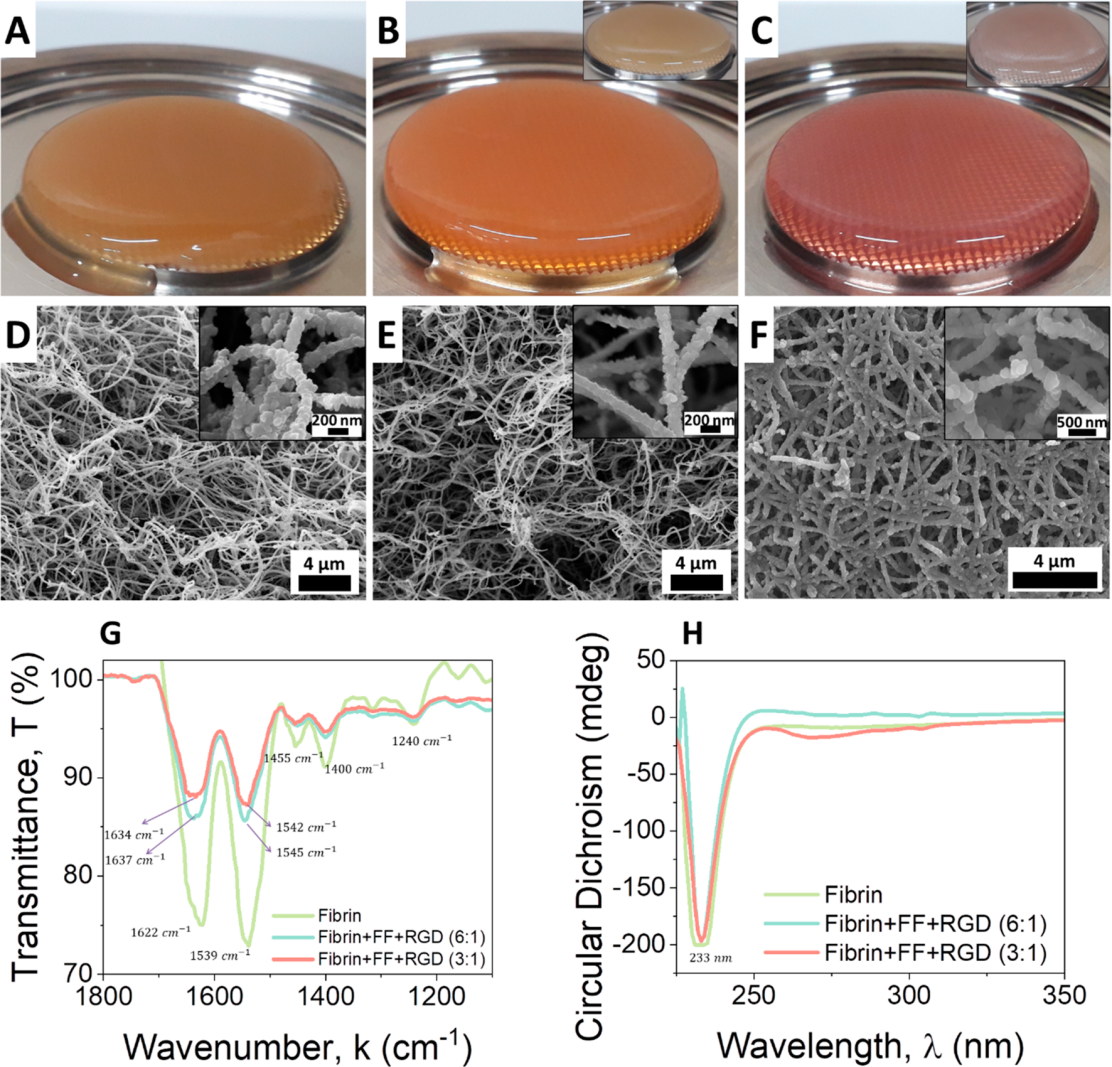
Appearance of the different samples analyzed in this work. (A) Fibrin hydrogel; (B) fibrin-Fmoc-FF hydrogel, 6:1 ratio (inset: fibrin:Fmoc-FF + Fmoc-RGD, 6:1 ratio); (C) fibrin-Fmoc-FF hydrogel, 3:1 ratio (inset: fibrin:Fmoc-FF + Fmoc-RGD, 3:1 ratio); (D) SEM image of fibrin hydrogel; (E) SEM image of fibrin:Fmoc-FF + Fmoc-RGD, 6:1 ratio; (F) SEM image of fibrin:Fmoc-FF + Fmoc-RGD, 3:1 ratio; (G) FTIR and (H) CD of hydrogel samples.

Considering the excellent biocompatibility properties of fibrin hydrogels, we decided to prepare mixtures with Fmoc-peptides, using small proportions of these peptides as compared to fibrin, but high enough to produce an improvement in their mechanical properties. Fibrin hydrogels were prepared from human plasma containing fibrinogen and a solution of tranexamic acid, DMEM (Dulbecco’s modified Eagle’s medium) and CaCl_2_ (see Experimental Section for more details), as described elsewhere.^50^ Composite hydrogels were formed after adding a solution of Fmoc-peptides sodium salt to the solution of fibrin precursor. Fmoc-peptides self-assembly was triggered by the presence of CaCl_2_.^26,29^ We selected two proportions (see Experimental Section) in which the volume ratios of the two solutions (fibrin precursors:Fmoc-peptides) were 6:1 and 3:1. When Fmoc-RGD was incorporated, the proportion between Fmoc-FF and Fmoc-RGD was always 7:3, because Ulijn et al. have shown that this proportion is enough to improve the mechanical properties and bioactivity of the resulting gels.^33^ The combination of fibrin precursors from human plasma and Fmoc-peptide solutions in both cases gave rise to homogeneous hydrogels that were significantly different to the naked eye than the gels obtained with fibrin alone (see Figure 2A–C). Hydrogels containing Fmoc-FF-peptide appeared more swollen as the amount of peptide increased from 6:1 (Figure 2B, inset includes Fmoc-RGD) to 3:1 (Figure 2C; inset includes Fmoc-RGD). In order to understand the type of interaction between fibrin and Fmoc-peptides, we analyzed the samples by scanning electron microscopy (SEM) (Figure 2D–F). Hydrogels obtained only with fibrin showed a dense mesh of intertwined fibers of several micrometers in length and an average diameter of 143 ± 4 nm (Figure 2D). These fibers appeared very amorphous, as they were formed by the aggregation of multiple small spheroidal fragments (see Figure 2D, inset). The analysis of the hydrogels made by the combination of fibrin:Fmoc-peptides at ratio 6:1 showed an aspect very similar to that of the fibrin gels (Figure 2E). The fibers were morphologically identical, showing the same amorphous pattern. The average fiber diameter in this mixture was also very similar (133 ± 3 nm). Nevertheless, the mixture fibrin:Fmoc-peptides 3:1 was clearly different. In this case, samples showed a denser mesh of fibers of much larger diameters (218 ± 5 nm). Similarly to the other samples, these broader fibers had the same amorphous appearance, although in this case, the small spheroidal aggregates were also much larger. It has been reported that when two different peptides co-assemble to form supramolecular aggregates, the morphology of the resulting co-aggregates can be similar to those of the individual peptides, but they can also be very different.^52−56^ In any case, a signature of co-assembly is the formation of a unique or preferential type of morphology that can be modified by varying the ratio of the components, as observed in this case.

Further analysis of the secondary structure of these hydrogels as well as the capacity of fibrin and peptides to interact with each other was studied by Fourier transform infrared spectroscopy (FTIR) (Figure 2G) and circular dichroism (CD) (Figure 2H) (HT spectra, Supporting Information Figure S1). FTIR spectroscopy of fibrin gel and fibrin-peptide hydrogels showed very similar spectra (Figure 2G). Fibrinogen, fibrin clots, and fragments of it have been studied in detail previously by FTIR spectroscopy.^57,58^ Although it is difficult to ascertain without ambiguity due to the overlap of amide bands, some works have estimated that fibrin clots are mainly composed of 30% α-helix, 40% β-sheets, and 30% turns. Amide I bands usually centered around 1650 cm^–1^ are generally assigned to α-helix, while bands around 1630 cm^–1^ correspond to β-sheet structures. Fmoc-dipeptide gels have also been characterized by FTIR. These peptides are usually arranged in antiparallel β-sheet secondary structures presenting a strong amide I band around 1630 cm^–1^.^17,26,59^ Antiparallel β-sheet structures usually have another amide I band around 1695 cm^–1^, although a study has shown that this band could correspond to the stacking of the carbamate group.^60^ Amide II bands (1580 to 1520 cm^–1^) are also sensitive to protein secondary structure and are related with N–H bending vibrations but also with aromatic amino acids side chains and COO^–^ stretching, and therefore, it is more difficult to infer secondary structures from this band. The spectra of fibrin gel (Figure 2G) presented two strong amide I and II bands centered at 1622 and 1539 cm^–1^, respectively. In this case, the appearance of the amide I band at 1622 cm^–1^ suggests that fibrin in these gels is mainly arranged in β-sheets. This can also be corroborated with the appearance at 1240 cm^–1^ of the amide III band.^57^ The incorporation of Fmoc-FF and Fmoc-RGD into the structure of fibrin gives rise to a blue shift of the amide I and II bands that now appear approximately at 1635 and 1545 cm^–1^. This shift in the bands must be due to an increase of intermolecular H bond formation mediated by Fmoc-peptides,^61^ but also to an overlap of the amide I and II bands of the Fmoc-peptides that appear at those wavelengths. In the same way, the CD spectra of the fibrin-peptide hydrogels looked all almost identical with the spectra of the fibrin hydrogel alone (Figure 2D). In all cases, the spectra presented a single negative band in the far ultraviolet region around 233 nm. The presence of this band, assigned to β-sheet structures, is well-documented for Fmoc-dipeptides^17^ and other hydrophobic oligomers.^62^ The reported CD spectra of fibrinogen shows two negative bands at 221 and 209 nm characteristic of α-helix.^63^ CD of fibrin has been more difficult to report due to the opacity of the samples, although spectra of some works show the presence of a single band with a negative maximum centered at 220 or 230 nm, similar to our results.^64^ Fmoc-dipeptides usually have another characteristic band in the near-ultraviolet region (270–320 nm) corresponding to (π–π* transition) which indicates superhelical arrangements formed by the Fmoc groups.^65^ This band is less intense in mixtures of fibrin:Fmoc-peptides (3:1) and is practically lost in 6:1 mixtures. The decrease in this band may suggest that the interaction of fibrin with peptides is also mediated by aromatic interactions disrupting the usual supramolecular arrangement of the Fmoc-peptides.

Therefore, the analysis of the data obtained by SEM, FTIR, and CD suggests that, indeed, fibrin and Fmoc-peptides are interacting with each other, giving rise to co-assembled fibrils that adopt a preferential β-sheet arrangement.

### Mechanical Evaluation of the Hydrogels

**Figure 3:**
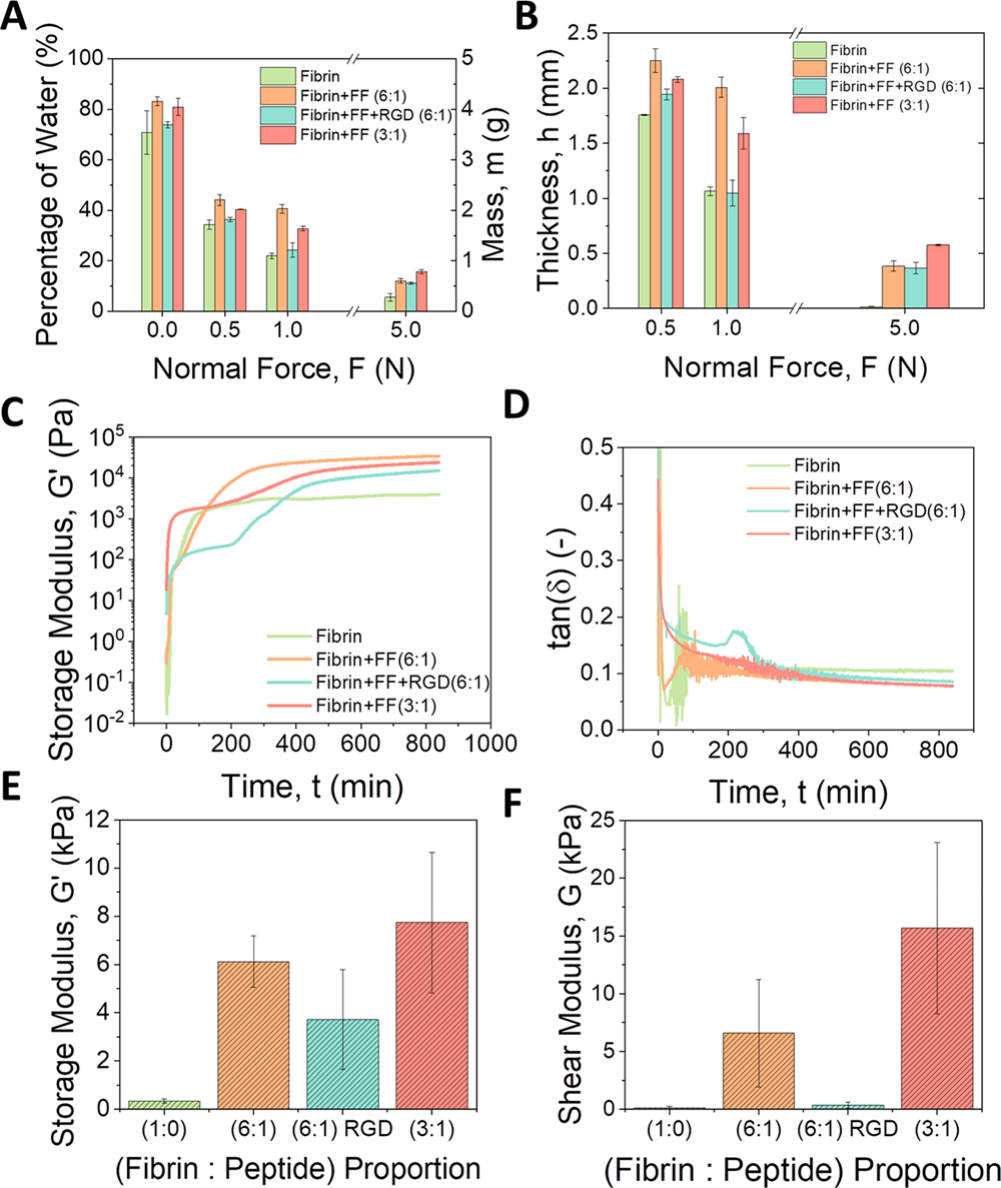
Biomechanical properties of the different hydrogels. (A) Percentage of water content and mass; (B) thickness of the hydrogels after the application of a normal force; (C) viscoelastic moduli (*G*′ and *G*″) as a function of time during the gelation kinetics; (D) loss tangent as a function of time during gelation starting from mixtures of pregel reagents; (E) mean values of storage modulus (*G*′) within the LVR for nanostructured hydrogels; (F) shear modulus (*G*) for nanostructured hydrogels.

A key property of hydrogels is their ability to retain water, even when subjected to mechanical stresses. With the aim of assessing the gel porosity (i.e., connected to water retention) and a rough estimation of the resistance to compression of the polymer structure, we studied the initial mass of pristine hydrogels, and the mass and thickness of the hydrogels after the application of different normal forces. The initial mass of hydrogels containing Fmoc-peptides was slightly higher than those of only fibrin (Figure 3A), which indicates a larger retention of water and, thus, a higher porosity, for the same total amount of polymer. This difference in mass (higher mass for fibrin:Fmoc-peptide hydrogels than for fibrin hydrogels) became relatively larger after the samples were subjected to normal forces (Figure 3A). This indicates a higher resistance of the polymer structures to compression when Fmoc-peptides were included in the formulation, with respect to pure fibrin hydrogels. The thickness of the samples followed a similar trend (Figure 3B), since there is a relation of proportionality with mass (note that all samples had the same horizontal section). These results agree with the macroscopic appearance of the hydrogels (Figure 2A–C), which evidence that fibrin-peptide hydrogels presented a more swollen consistency than those of only fibrin.

Separately, we monitored the evolution of the storage (*G*′) and loss (*G*″) moduli as a function of time, in order to study the kinetics of gelation of the hydrogels. *G*′ gives a measure of the elastic energy of the sample, while *G*″ is related to the dissipation of viscous energy. In all cases, we observed a gel-like character (*G*′ > *G*″) at the very beginning of the experiments, with an initial very sharp increase of the viscoelastic moduli related to a fast strengthening of the gel structure, followed first by an inflection point and then by a second strong increase of viscoelastic moduli, until a final trend to saturate (plateau zone) at large time, indicating the saturation of cross-linking (Figures 3C and S2). Note at this point that Adams et al.^23^ found an inflection point in the curve of storage modulus vs time during gelation of self-sorting peptide gels. However, here we observed the inflection point even for pure fibrin gels, and thus, it cannot be due to a self-sorting mechanism. Furthermore, the shape of these curves of kinetic of gelation (viscoelastic moduli vs time) are similar for fibrin hydrogels and for fibrin:Fmoc-peptide hydrogels, which points toward a co-assembly mechanism. At low gelation times, the gels presented a weak gel character, with values of tan(δ) = *G*″/*G*′ in the range 0.1 to 1 (Figure 3D). However, fibrin hydrogels presented final values of tan(δ) ≈ 0.1, typical of hydrogels in the frontier between weak and strong gels, whereas fibrin:Fmoc-peptide hydrogels presented values of tan(δ) < 0.1, corresponding to a typical behavior of strong gels (Figure 3D). In addition, both the curves of *G*′ and *G*″ vs time, as well as these of the loss tangent vs time corresponding to the kinetics of gelation, evidence that the fibrin hydrogels complete gelation was faster compared to those containing Fmoc-peptides in their composition. Interestingly, some previous works have reported slow gelation kinetics for co-assembly of structurally different peptides,^52,56^ which further supports the hypothesis of co-assembly of fibrinogen and Fmoc-peptides, given that they are structurally very different. Furthermore, the SEM analysis discussed above evidence thicker fibers in the case of fibrin:Fmoc-peptides hydrogels at ratio 1:3 than in fibrin hydrogels, which agrees with the hypothesis of co-assembly.

We also characterized the mechanical properties of hydrogels under oscillatory shear after complete gelation. From amplitude sweeps (*G*′ and *G*″ vs strain amplitude, γ, at constant oscillation frequency, *f*), trends typical of polymer hydrogels were obtained (Figure S3A). As observed, these curves are characterized by values of *G*′ and *G*″ almost independent of strain amplitude, with *G*′ >*G*″ at γ < 0.1, which defines the linear viscoelastic region (LVR). This region is followed by a steep drop of *G*′, accompanied first by a slight increase of *G*″ up to a maximum that defines the yielding point, and an ulterior decrease of *G*″. Destruction of the polymer network takes place in this region and the strain for which the crossover of *G*′ and *G*″ occurs defines the limit of the gel-like behavior, above which the polymer network is no longer percolating. The gel-like character of the samples was also corroborated by the mechanical spectra (*G*′ and *G*″ within the LVR vs frequency) that presented values of *G*′ > *G*″ almost independent of frequency, as expected for cross-linked polymeric networks (Figure S3B).

Although fibrin:Fmoc-peptide hydrogels showed *G*′ values of 1 order of magnitude higher than those of fibrin hydrogels in the gelation kinetics measurements, where the final volume and polymer concentration were maintained in all cases (Figure 3C), relating to the values of storage modulus within the LVR, differences between samples were small for pristine (not compressed) hydrogels (Figure S3). However, it is impossible to establish a suitable comparison in this case as the pristine hydrogels had different mass and thickness for the same applied normal force (Figure 3A). Thus, in order to obtain properly comparable results for the mechanical properties of the hydrogels, a nanostructuration process (see Experimental Section) was made by the application of a plastic compression which enabled one to obtain hydrogels with the same mass and thickness, and therefore, polymer content, in all cases.^66^ Then, after plastic compression (nanostructuration) fibrin:Fmoc-peptide hydrogels demonstrated *G*′ values of about an order of magnitude larger than those of only fibrin for similar total volume (i.e., similar polymer content—see Table 1; note that the initial volume of fibrin:Fmoc-peptide hydrogels was larger) (Figure 3E). Furthermore, the value of *G*′ increased as the amount of Fmoc-peptides increased, going from 6.1 ± 1.1 kPa values for mixtures of fibrin:Fmoc-peptides at 6:1 ratio to 7.7 ± 2.9 kPa for mixtures at 3:1 ratio. In addition, the shear modulus (*G*), defined as the slope of the curves of stress against strain in steady deformation, went from 0.13 kPa for fibrin nanostructured samples to 16 kPa for fibrin:Fmoc-peptide gels, that is, 2 orders of magnitude higher (Figure 3F). Therefore, it can be concluded that fibrin:Fmoc-peptide hydrogels exhibited strongly improved mechanical properties compared to the hydrogel containing fibrin alone. This large enhancement of the storage modulus and shear modulus of fibrin:Fmoc-peptide gels with respect to the hydrogels constituted by fibrin supports the hypothesis of co-assembly of these gelators. This hypothesis seems especially plausible since Fmoc-FF and Fmoc-RGD peptides form extremely weak gels at the concentrations used in this work (Figure S2B), and thus, it does not seem likely that a mechanism other than co-assembly of fibrin and peptides could be responsible for the significant enhancement of the mechanical properties of fibrin:Fmoc-peptide hydrogels. It has been reported that co-assembly of dipeptides gave rise to a large enhancement of the mechanical properties of peptide hydrogels.^23,30,31,52^

**Table 1:** Volume Proportions of the Components for Hydrogel Preparation

Sample (5 mL)	Vol of fibrin precursor (mL)	Vol of peptide solution^a^^b^ (μL)	Vol of additional DMEM (μL)	Total polymer/peptide content (mg/mL)
Fibrin	5	0	0	1.5
Fibrin–peptide (6:1)	4.25	357	357	2
Fibrin–peptide (3:1)	3.75	625	625	2.5

a Peptide concentration was 20 mM in all cases.

b Ratio of Fmoc-FF to Fmoc-RGD was 7:3.

### In Vitro Structural Stability of the Hydrogels

**Figure 4:**
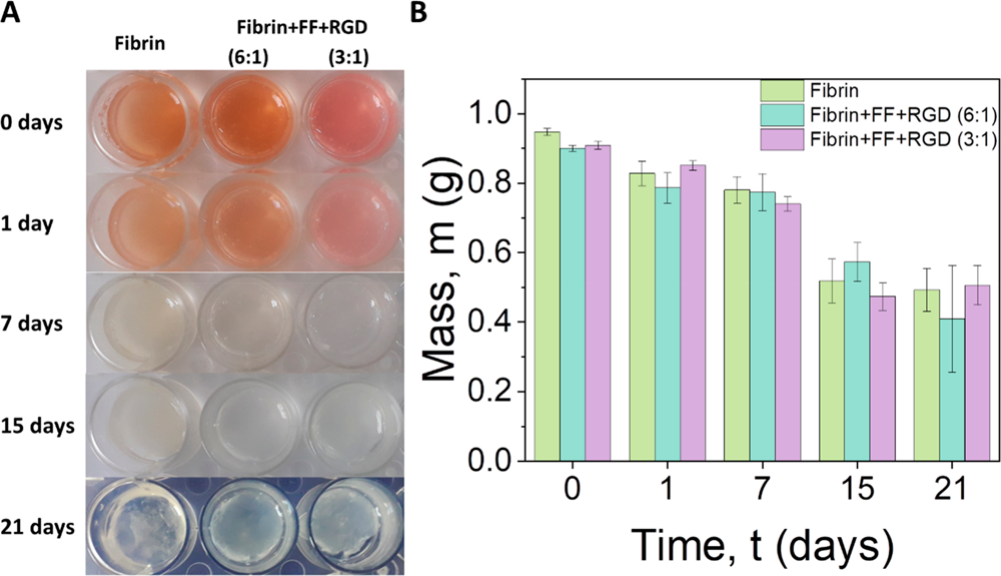
Degradation behavior. (A) Representative images of acellular hydrogels at selected periods after preparation; (B) mass of the hydrogels as a function of time.

As noted in the in vivo experiments, hydrogels were reabsorbed in the histological analysis of the implant areas after 1 and 3 weeks from the injection in the animals. To further investigate the degradation behavior, we prepared acellular hydrogels and monitored the changes in mass and integrity over time when immersed in PBS. All samples were progressively degraded and decreased in mass over time, showing similar integrity, independently of the inclusion of the peptides at different ratios (Figure 4). However, they remained stable after 21 days, in contrast with the in vivo observations. Here, it must be considered that the hydrogels, when implanted in vivo, were subjected not only to biodegradation by host living cells but also to compressive and tensile forces due to the movement of the animals, while no external forces disturbed them in the present test. Therefore, the degradation of the hydrogels is faster in the animals than in the laboratory conditions, and the presence of the peptides at different ratios does not have an influence on this process.

### Ex Vivo Evaluation of Hydrogel Biocompatibility

**Figure 5:**
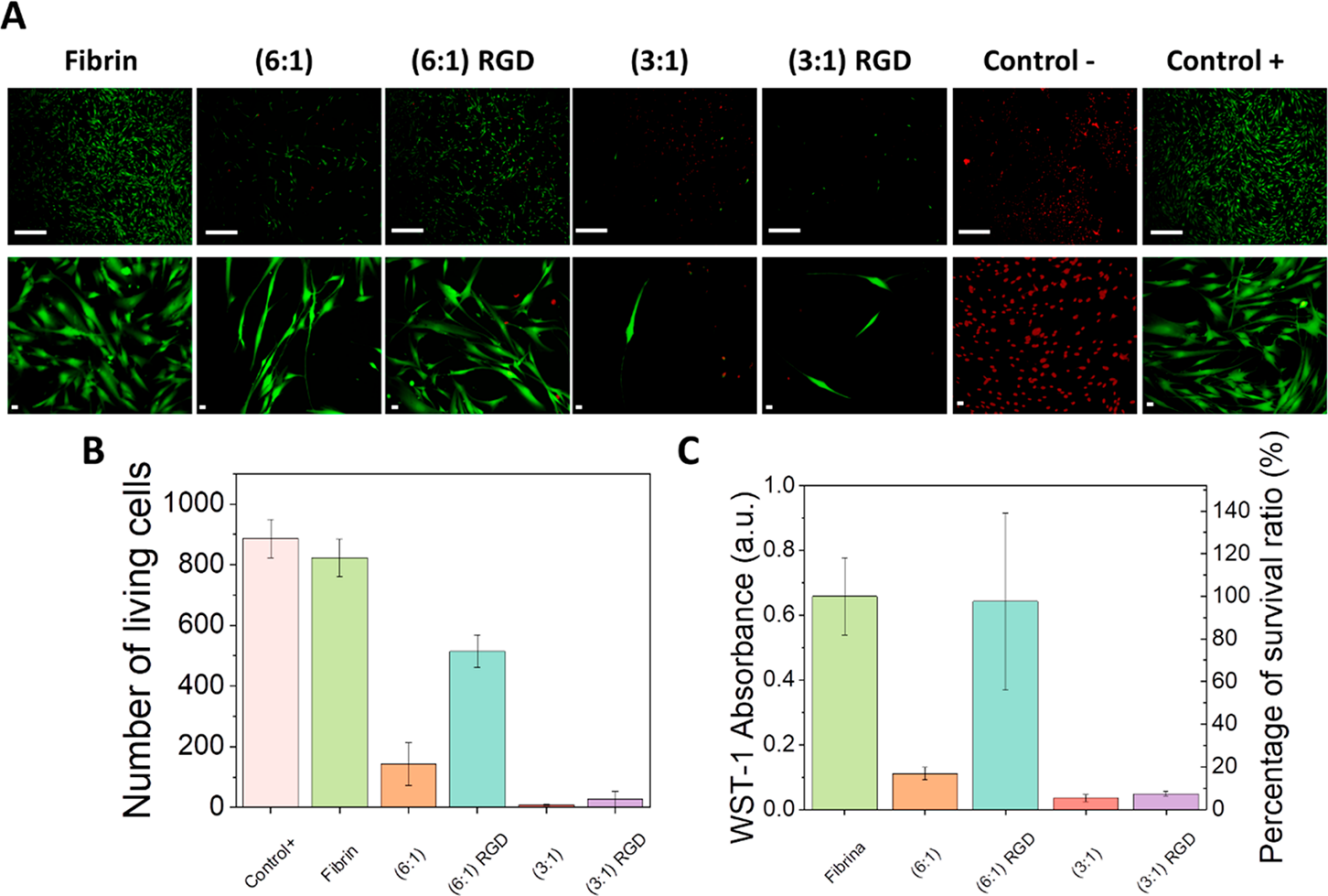
Analysis of cell viability. (A) Microscopic images of cells from live/dead assays (scale bar, 200 μm); number of living cells from (B) live/dead assays and C) WST-1 assays.

We then sought to investigate cell proliferation in the presence of the different hydrogels to get a measure of the ex vivo biocompatibility of each biomaterial. For this aim, we carried out 2D cell cultures of human fibroblasts and put them in contact with the hydrogel samples using culture inserts for 48 h. Fluorescent microscopy pictures obtained after staining the cells with the live/dead assay reagent showed that the cells in contact with the fibrin gel prevalently appeared green-stained and elongated, thus confirming that they were alive and functional (dead cells are stained in red in the live/dead assay), similar to the positive control group (Figure 5A). Furthermore, quantification of the number of living cells confirmed that there were no statistically significant differences (*p* < 0.05) between the fibrin hydrogels and the positive control (Figure 5B). This is not surprising, since fibrin is a natural polymer successfully used in tissue engineering.^50,67,68^ Combination of fibrin and Fmoc-FF resulted in a dramatic reduction of the number of living cells (Figure 5A,B) with the presence of some red-stained cells in the fluorescence microscopy experiments (Figure 5A). The number of viable cells was further reduced when the proportion fibrin:Fmoc-FF varied from 6:1 to 3:1, thus pointing at Fmoc-FF as responsible for the decrease in cell proliferation. Such a decrease indicates that cell viability and proliferation are seriously compromised in fibrin-Fmoc-FF hydrogels, which in principle, could not compensate for the improvement of the mechanical properties observed in these samples. However, this trend can be successfully reversed upon small additions of Fmoc-RGD. Indeed, the number of living and elongated cells almost triplicated in the presence of Fmoc-RGD, although the number of living cells was still slightly smaller than for fibrin gels alone. This result, together with the improvement of the mechanical properties obtained for the fibrin:Fmoc-FF + RGD sample (6:1), makes it an excellent candidate for diverse biomedical applications.

To further analyze the biocompatibility of the hydrogels, we carried out WST-1 assays based on the colorimetric transformation of tetrazolium salt (WST-1) to formazan driven by the mitochondrial dehydrogenase activity of living cells, which is directly proportional to the number of viable (i.e., metabolically active) cells. As described in the previous paragraph for the live/dead assay, a decrease in absorbance, connected to a decrease in the number of viable cells, was measured for fibrin-Fmoc-FF hydrogels compared to fibrin gels, and this decrease was stronger when the relative amount of Fmoc-FF peptide was increased (Figure 5C). Again, cell viability was improved upon Fmoc-RGD addition to the formulation. Remarkably, results demonstrate that no statistically significant (*p* < 0.05) differences exist between fibrin hydrogels and fibrin:Fmoc-FF peptide (6:1) hydrogels containing Fmoc-RGD (Figure 5C), confirming that cells are able to proliferate and remain viable in contact with the hydrogels. Thus, the inclusion of a small amount of peptides in the hydrogel formulation based on a careful choice of peptide composition/proportion creates a good environment for cell viability and a great enhancement on their mechanical properties. Note that not only biocompatibility but also an adequate internal structure and mechanical properties are considered essential characteristics of biomaterials.^69^ The positive effects of RGD peptides on cell viability were previously demonstrated for several types of biomaterials and RGD has been used to modulate and improve the biological behavior of biomaterials used in biomedicine.^33^ Most likely, the high sequence homology of RGD peptides with key proteins involved in cell proliferation, migration, and attachment, such as fibronectin and vitronectin, could explain their biological effects.^33^

### In Vivo Biocompatibility and Biosafety of the Hydrogels

**Figure 6:**
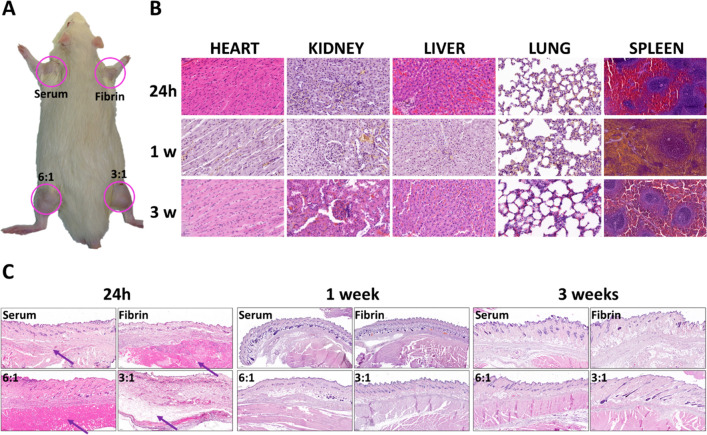
(A) Implant sites in the animals used for the in vivo experiments. Histological analysis (hematoxylin and eosin staining) of the (B) five systemic organs in rats (heart, kidney, liver, lung, and spleen) and (C) implant site in animals followed for 24 h, 1 week, and 3 weeks after in vivo grafting. Photographs are representative images of each experimental condition. Location of the injected materials is highlighted with arrows. Scale bars: 100 μm in panel A and 500 μm in panel B.

We also tested the time-dependent biosafety of the hydrogel samples in vivo by injecting them subcutaneously in rats. None of the animals died during the experiments, and they did not show any signs of systemic alterations (see Table S1, for hematological testing results). There was no evidence of side effects or changes in the body weight compared to control animals. Histological analyses of key vital organs (spleen, liver, kidney, heart, and lungs) showed a normal histological pattern and structural stability without any signs of damage or cytotoxicity after the 3 week follow-up period (Figure 6A). In addition, our analysis of the relevant biochemical parameters in blood of the animals included in the study showed that all these parameters were within normal ranges after 1 and 3 weeks of follow-up, with nonsignificant differences with control animals. As these parameters were directly related with the physiology of the key vital organs, these results confirm that the injection of the different biomaterials was not associated with a relevant alteration of the major organs of the animals and that these organs could therefore maintain their physiological functions in vivo (see Table S2 for biochemical parameters in blood).

When the injection site was analyzed histologically, we found that the different materials were restricted to the subcutaneous layer and showed a homogeneous fibrillar appearance 24 h after the implant (Figure 6B). There were no signs of side effects derived from the injection in any of the experimental groups, which supports the cell viability, metabolic activity, proliferation, and functionality of the ex vivo biocompatibility analyses (Figure 6B). However, the grafting area was slightly edematized 24 h after the injection in the fibrin-Fmoc-FF 3:1 group, although other side effects were not detected (Figure 6B). This could be related to a worse performance of this sample in terms of cell viability observed in Figure 4. After 1 and 3 weeks of the injection, all injected hydrogels appeared almost reabsorbed, with no signs of inflammatory response, fibrosis, necrosis, infection, or malignant transformation. These results suggest that these hydrogels could work as excellent platforms for short-term biomedical applications such as injectable vehicles for transport and release of cells/specific molecules, where a rapid degradation and release from the injection site is desired. Indeed, even the tougher fibrin-Fmoc-FF samples were easily injected as shown in Video S1. Future studies should evaluate the potential usefulness of these hydrogels in other applications, such as wound healing.

## Conclusions

In summary, we report a simple protocol to obtain composite fibrin:Fmoc-peptide hydrogels for regenerative medicine. The formation of fibrin hydrogels, triggered by fibrinogen self-assembly, in the presence of Fmoc-peptides, showed that these peptides integrated into the fibrin matrix through a co-assembly process. This resulted in composite hydrogels with improved mechanical properties while retaining their porosity and reticular structure. The inclusion of Fmoc-RGD significantly improved the ex vivo biocompatibility properties, reaching cell viability values similar to those of fibrin hydrogels, which is remarkable considering the lower proportion of fibrin in the composites. Composite hydrogels could also jellify in situ, allowing the administration of pregel mixtures by injection. In vivo, results showed that the composites behaved similarly to fibrin hydrogels, being completely resorbable without causing any inflammatory response. This offers the possibility of using these composite hydrogels as cell carriers, wound healers, and bio-adhesives, as well. Fmoc-FF and Fmoc-RGD are simple organic molecules that can be easily synthesized using a solid phase protocol. In particular, these peptides are commercially available at an affordable price. The possibility of adjusting the properties of fibrin hydrogels with these peptides provides an approach to prepare biocompatible and biofunctional fibrin hydrogels at a lower cost. Furthermore, fibrin hydrogels can be modified by other bioactive peptide gelators offering the possibility to broaden their applicability in different biomedical fields, which is worth further study.

## Experimental Section

### Reagents and Materials

N-Fluorenylmethoxycarbonyl diphenylalanine (Fmoc-FF) and *N*-fluorenylmethoxycarbonyl arginylglycylaspartic acid (Fmoc-RGD) peptides were purchased from LifeTein (USA) and SynPeptide (CHN), respectively, and were used without further purification. Sodium hydroxide (NaOH), calcium chloride (CaCl_2_), phosphate buffered saline (PBS), and Dulbecco’s modified Eagle’s medium (DMEM) were provided by Sigma-Aldrich, USA. The tranexamic acid used as an antifibrinolytic agent (Amchafibrin) was purchased from Fides-Ecofarma, Valencia (Spain).

### Preparation of Peptide Solutions

The Fmoc-FF solution was prepared following the next protocol. The desired amount of Fmoc-FF was weighed, and Milli-Q water was added in order to obtain a final suspension of 20 mM concentration. The resulting suspension was sonicated in a cold ultrasonic bath (AL04-03-230) until a homogeneous suspension was obtained after approximately 1 h. Then, a solution of 0.5 M NaOH was added dropwise (drop volume of 10 μL) using a micropipette and sonicating for 2 min just after the addition of each drop. A clear solution was obtained at a pH of approximately 10.5.

On the other hand, a Fmoc-RGD solution was prepared by adding the proper amount of Fmoc-RGD powder to Milli-Q water and stirring until a clear 20 mM solution was obtained.

For the preparation of hydrogels containing the biological ligand, Fmoc-FF:Fmoc-RGD solutions were mixed at a 7:3 ratio.

### Preparation of Uncompressed and Nanostructured Fibrin–Peptide Hydrogels

For the preparation of uncompressed fibrin-peptide hydrogels, we proceeded as it follows. Fibrin hydrogels were prepared by mixing 3.8 mL of human plasma, 875 μL of DMEM (Dulbecco’s modified Eagle’s medium), 75 μL of Amchafibrin, and 250 μL of a 2% CaCl_2_ solution in PBS (to promote fibrin gelation) for a final volume of 5 mL. In the case of fibrin-peptide hydrogels, the precursor of fibrin hydrogels (i.e., same composition as for fibrin gels) was mixed with the peptide solution and an additional amount of DMEM (to promote Fmoc-FF gelation) equal to the volume of peptide (see Table 1). Finally, the mixture was left overnight at 37 °C to ensure complete gelation. As a control experiment, peptide hydrogels at the studied volume ratios were prepared by the addition of Milli-Q water instead of the fibrin precursor (Figure S2B), following the same proportions from Table 1.

To prepare nanostructured hydrogels, they were subjected to plastic compression techniques as described previously.^49^ For this purpose, the hydrogels were placed between a pair of nylon filter membranes of 0.22 μm pore size (Merck-Millipore, Darmstadt, Germany) and a pair of absorbent pieces of paper. Then, the hydrogels were compressed for 3 min under uniform mechanical pressure of ∼520 Pa, homogeneously distributed.

#### Physicochemical Characterization of the Hydrogels

##### Scanning Electron Microscopy

We analyzed the microstructure of hydrogels by scanning electron microscopy (SEM) by means of a FEI Quanta 400 SEM (FEI Co., Hillsboro, OR, USA). The hydrogel specimens were prepared according to a well-established protocol in order to subject them to CO_2_ critical point drying^70^ and coat them with Au–Pd (ion sputtering method).

##### Fourier Transform Infrared Spectroscopy

We recorded Fourier transform infrared spectroscopy (FTIR) spectra by using a PerkinElmer Two FTIR ATR spectrometer (PerkinElmer Co., Waltham, MA, USA). The hydrogels were compressed onto the diamond crystal, and the spectra were scanned over the wavenumber range from 4000 to 450 cm^–1^.

##### Circular Dichroism

The hydrogels were gelled into a 0.1 mm quartz cell (Hellma 0.1 mm quartz Suprasil) and the circular dichroism (CD) spectra were measured using a Jasco J-815 spectropolarimeter (Jasco Co., Japan) equipped with an air cooled 150 W xenon lamp. A constant temperature of 20 °C was maintained during the measurements using a PFD-425 Peltier controller. The spectra were recorded from 200 to 350 nm with a step of 1 nm and 0.5 s of integration time per step. For each experimental condition, we took the average value of 100 measurements.

#### Mechanical Evaluation of the Hydrogels

##### Gel Kinetics

We investigated the gel kinetics of hydrogels by means of rheological measurements, using a Haake MARS III controlled-stress rheometer (Thermo Fisher Scientific, Waltham, MA, USA) provided with a double cone–plate sensor of 60 mm of diameter and 2° apex angle (sensor DC60/2° Ti L). With this aim, we followed the protocol for the preparation of the hydrogels described above and poured the mixture in the measuring system of the rheometer. Then, we subjected the gelling sample to oscillatory shear strain of fixed frequency (1 Hz) and strain amplitude (γ_0_ = 0.001), monitoring the resulting viscoelastic moduli as a function of time and at a constant temperature of 37.0 ± 0.1 °C. The strain amplitude used in our work was low enough to ensure that the building of the gel microstructure was unperturbed.

##### Characterization of the Mechanical Properties of the Hydrogels

We characterized the mechanical properties of the hydrogels under oscillatory shear strains by using a Haake MARS III controlled-stress rheometer (Thermo Fisher Scientific) provided with a double plate sensor of 35 mm of diameter and rough surfaces to avoid wall slip (sensor P35 Ti L S serrated, Thermo Fisher Scientific). Characterization was carried out at a constant temperature of 37.0 ± 0.1 °C. First, we subjected the hydrogels to amplitude sweeps, for which the frequency of oscillation was kept at 1 Hz and the amplitude of the oscillatory strain, γ_0_, was increased stepwise from 0.0001 to 2. From these measurements we obtained the values of the storage (*G*′) and loss (*G*″) moduli as a function of γ_0_. Afterward, we performed frequency sweep tests, for which the amplitude of the shear strain was fixed at γ_0_ = 0.001, and the frequency of oscillation was increased stepwise from 0.1 to 16 Hz. From these measurements we obtained the values of *G*′ and *G*″ as a function of the frequency (i.e., the mechanical spectra) of the hydrogels. In order to analyze the shear modulus (*G*) of these hydrogels, we applied shear stress sweeps from 0.1 to 300 Pa.

A fresh sample was used for each test (amplitude and frequency sweeps) and experimental conditions, and measurements for at least three different aliquots of the same experimental condition were conducted. In this work we provide the corresponding mean values and standard errors of the measurements.

#### Cell Viability Assessment

To test cell viability, first, a dispersion of primary human skin fibroblasts derived from healthy donors and cultured in DMEM was seeded on the bottom of culture wells at a density of 15 × 10^3^ cells per culture well and kept in culture for 24 h. Then, the hydrogels were put in contact with the cultured cells using culture inserts for 48 h. Finally, hydrogels were removed, and cells were analyzed using Live/Dead Cell Viability Assays (Invitrogen, Waltham, MA, USA) and water-soluble tetrazolium salt-1 (WST-1) assays (Roche, Basel, Switzerland), following the manufacturer’s instructions and previous studies (PMID: 36082161). Briefly, to perform the Live/Dead Cell Viability Assay (LD), culture wells with cells seeded on bottom were rinsed in PBS three times (5 min each) and then incubated with the working solution for 30 min. Cells were then rinsed in PBS and analyzed using a Nikon Eclipse Ti fluorescence inverted microscope (Nikon, Tokyo, Japan) equipped with a Nikon DXM 1200c Digital Camera (Nikon). For WST-1, after cell incubation and once the hydrogels and the medium were removed, the culture wells were rinsed with PBS and incubated with the WST-1 (Cell Proliferation Reagent WST-1, Roche Diagnostics, Mannheim, Germany) working solution for 4 h at 37 °C. In all cases, hydrogels without cells seeded on the bottom of the culture well were used as controls. In addition, cells seeded on chamber slides without contact with hydrogels were used as positive technical controls (100% cell viability), while cells seeded on chamber slides and incubated with 2% Triton X-100 were used as technical negative controls (0% cell viability). For the statistical analysis, three samples of each condition were studied.

#### In Vivo Biocompatibility

Biocompatibility of the hydrogels was assessed in 9 adult male Wistar laboratory rats. The animals were deeply anesthetized with ketamine and acepromazine, and four subcutaneous injections were made in each rat with 500 μL of (i) saline solution only (used as a control), (ii) fibrin hydrogel, (iii) fibrin-Fmoc-FF+Fmoc-RGD hydrogel, 6:1 ratio, and (iv) fibrin-Fmoc-FF+Fmoc-RGD hydrogel, 3:1 ratio. Each animal received one injection of each of the four precursor materials in different parts of the dorsal area, close to the origin of the four limbs. Animals were euthanatized by intraperitoneal injection of a euthanasia solution (Eutanax 200, Fatro Ibérica, Barcelona, Spain) after 1, 7, or 21 days of follow-up, and the main organs (heart, kidney, liver, lung, and spleen), along with the four injection areas, were removed and analyzed histologically. Blood samples were obtained from each study animal after 1 and 3 weeks and controls just before administering the euthanasia solution, and the following parameters were determined in plasma: ALP (U/L), ALT (U/L), amilase (U/L), AST (U/L), direct bilirubin (mg/dL), total bilirubin (umol/L), creatine kinase (CK; U/L), creatinine (mg/dL), GGT (U/L), glucose (mg/dL), LDH (U/L), lipase (U/L), uric acid (mg/dL), and urea (mg/dL).

For the statistical analyses, the data obtained for each study group were compared with the t-student statistical. In all cases, *P* values below 0.05 were considered statistically significant.

Animal experimentation was approved by the Animal Experimentation Ethics Committee (Comité de Ética y Experimentación Animal, CEEA), protocol codes 19/04/2021/053 (date of approval Mar. 21, 2021) and 08/07/2019/123 (date of approval Sep. 10, 2019).

#### Structural Stability Studies

For the degradation experiments, the hydrogels (three samples of 1 mL per experimental condition) were prepared in Eppendorf tubes and 500 μL of PBS was added and stored at 37 °C in a laboratory oven. The PBS was removed and the mass of the hydrogels was measured every 24 h. To study the integrity, the hydrogels (three samples per experimental condition) were prepared in well plates, which were then immersed in 500 μL of PBS and stored at 37 °C in a laboratory oven. Every 24 h, the PBS medium was changed, and we monitored the physical integrity of the hydrogels by direct observation and photography with a digital camera.

## Supporting Information Available

(Figure S1) HT spectra of CD reported in the main text; (Figure S2) gelation kinetics of the hydrogels; (Figure S3) rheological properties for fibrin and fibrin:Fmoc–peptides hydrogels; (Table S1) hematological testing results; (Table S2) biochemical parameters in blood (PDF)
Video S1 showing injectability of composite hydrogels (fibrin:Fmoc–peptide,s 6:1 ratio) (MP4)
