# Supplementary material for: Biocompatible Short-Peptides Fibrin Co-assembled Hydrogels
Source: ACS Appl Polym Mater. 2023 Feb 21;5(3):2154–65. doi: 10.1021/acsapm.2c02164 (PMC10013376; doi:10.1021/acsapm.2c02164)
Supplement: Supplementary file 1 — ap2c02164_si_001.pdf [file ap2c02164_si_001.pdf]

SUPPORTING INFORMATION

Biocompatible short-peptides fibrin co-assembled hydrogels

Cristina Gila-Vilchez,[a,d] Mari Carmen Mañas-Torres,[b,d] Óscar Darío García-García,[c,d] Alfredo Escribano-Huesca,[a] Laura Rodríguez-Arco,[a,d] Víctor Carriel,[c,d] Ismael Rodriguez,[c,d] Miguel Alaminos,[c,d] Modesto Torcuato Lopez-Lopez,[a,d*] Luis Álvarez de Cienfuegos[b,d*]*

[a] Universidad de Granada (UGR), Departamento de Física Aplicada, C. U. Fuentenueva, Avda. Severo Ochoa s/n, E-18071 Granada.

[b] UGR, Departamento de Química Orgánica, Unidad de Excelencia Química Aplicada a Biomedicina y Medioambiente (UEQ), C. U. Fuentenueva, Avda. Severo Ochoa s/n, E-18071 Granada, Spain.

[c] UGR, Department of Histology, Avenida de Madrid 11, 18012 Granada, Spain

[d] Instituto de Investigación Biosanitaria ibs.GRANADA, Av. De Madrid, 15, 18016, Granada, Spain.

* Corresponding authors: malaminos@ugr.es (M. Alaminos), modesto@ugr.es (M.T. Lopez-Lopez), lac@ugr.es (L. Álvarez de Cienfuegos)

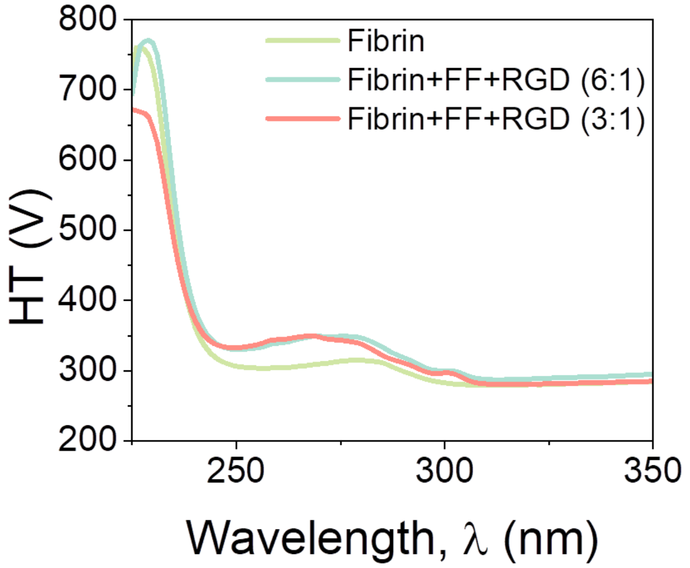

Figure S1. HT spectra of CD reported in the main text.

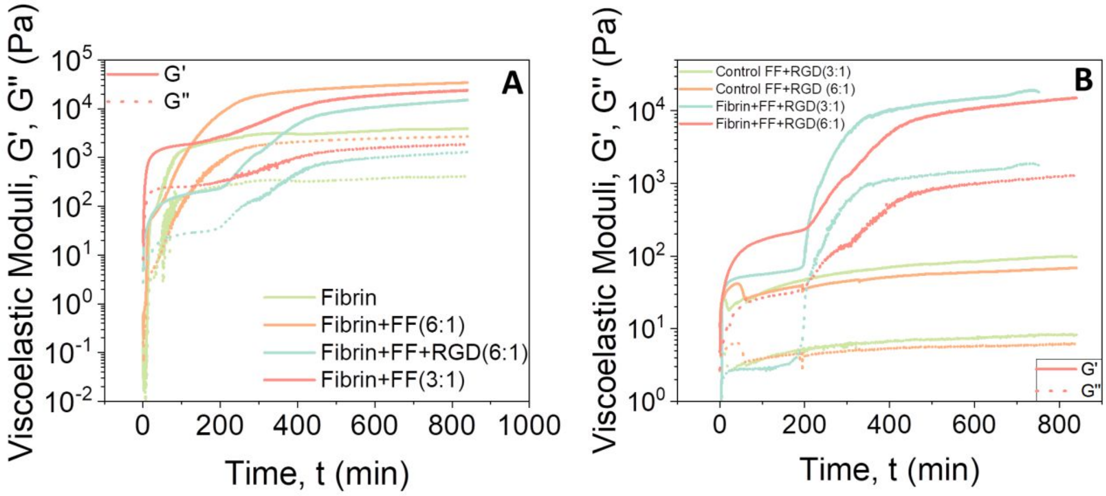

Figure S2. Gelation kinetics of the hydrogels. A) Viscoelastic moduli as a function of time for fibrin and fibrin-peptides hydrogels; B) Viscoelastic moduli as a function of time for fibrin-peptides and control peptides hydrogels at (6:1) and (3:1) ratios.

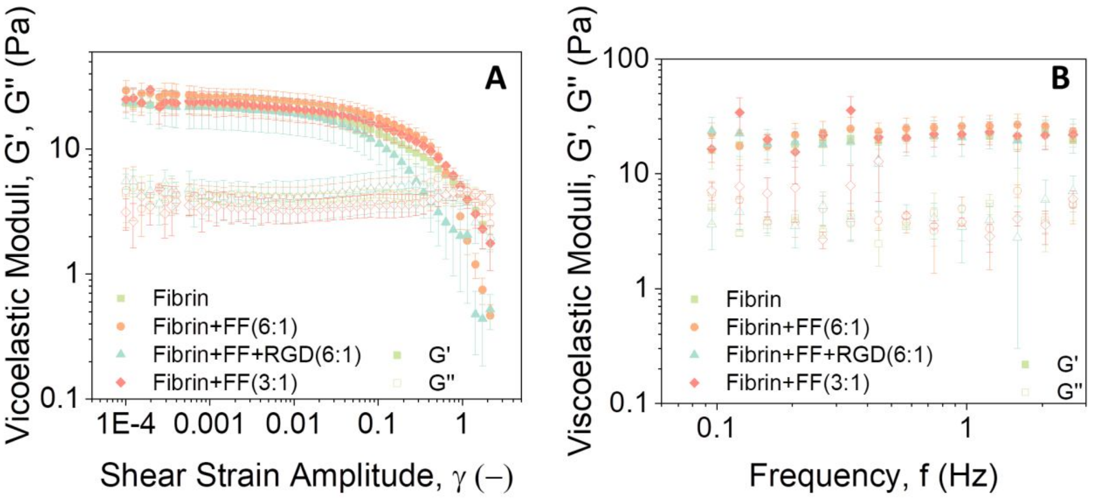

Figure S3. Rheological properties for fibrin and fibrin:Fmoc-peptides hydrogels. A) Viscoelastic moduli as a function of the shear strain amplitude for pristine fibrin and fibrin-peptides hydrogels; B) Viscoelastic moduli as a function of the oscillatory frequency for pristine fibrin and fibrin-peptides hydrogels. In both cases, the experiments were made after the application of a normal force equal to 1N.

Table S1. Average values of the main hematological parameters in blood of animals grafted with the different materials for 1 week (1W) and 3 weeks (3W). Note that each animal of the experimental group received an injection of the four materials at different parts of the dorsal area.

	RBC	HGB	HCT	MCV	MCH	MCHC	RDW_SD	RDW_CV	WBC	LYM#	MXD#	NEUT#	PLT	PDW	MPV	P_LCR
1W	$(7.4\pm1.0)\cdot10^6$	13.5 ±1.2	40 ±6	53.9 ±0.9	18.2 ±1.1	33.8±1.9	30.1±1.0	15±1	$(9.9\pm2.1)\cdot10^3$	$(8.5\pm1.5)\cdot10^3$	$(1.0\pm0.5)\cdot10^3$	400 ±170	$(6.0\pm4.5)\cdot10^5$	8.6 ±1.0	6.8 ±0.3	6.5±2.5
3W	$(7.84\pm0.20)\cdot10^6$	14.1 ±0.7	42.6 ±1.4	54.3 ±0.5	17.9 ±0.5	33.0±0.5	29.8±0.7	14.3±0.5	$(5.7\pm1.6)\cdot10^3$	$(4.7\pm1.1)\cdot10^3$	700 ±260	300 ±260	$(2.1\pm2.4)\cdot10^5$	14 ±5	9.2 ±1.8	28±18

Abbreviations:

RBC (/uL): red blood cell

HGB (g/dL): hemoglobin

HCT (%): hematocrit

MCV (fL): mean corpuscular volume

MCH (pg): mean corpuscular hemoglobin

MCHC (g/dL): mean corpuscular hemoglobin concentration

RDW_SD (fL): red blood cell distribution width-standard deviation

RDW_CV (%): red blood cell distribution width-coefficient of variation

WBC (/uL): white blood cell

LYM# (/uL): lymphocyte count

MXD# (/uL): mixed cell count

NEUT# (/uL): neutrophils count

PLT (/uL): platelet count

PDW (fL): platelet distribution width

MPV (fL): mean platelet volume

P_LCR (%): Platelet-large cell ratio

Table S2. Average values of the main biochemical parameters in plasma of animals grafted with the different materials for 1 week (1W), 3 weeks (3W) and control (CTR). Note that each animal of the experimental group received an injection of the four materials at different parts of the dorsal area.

	**ALP**	**ALT**	**Amilase**	**AST**	**Direct Bilirubin**	**Total Bilirubin**	**Creatine Kinase**	**Creatinine**	**GGT**	**Glucose**	**LDH**	**Lipase**	**Uric Acid**	**Urea**
CTR	109.0±0.7	43.2±0.4	1979±21	76.7±0.5	0.1	0.8±0.4	374±3	0.33±0.03	0	180±17	388±20	5.77±0.23	0.2	26.2±0.2
1W	96 ±11	95±65	1980±53	65±9	0.1	1.4±0.3	270±80	0.33±0.03	0	197.0±2.7	326±75	5.3±0.3	0.7±0.3	26.5±0.5
3W	134 ±17	46.6±2.1	2034±130	102±17	0.1	1.30±0.17	530±130	0.4	0	188±11	400±70	6.3±0.3	0.27±0.03	28.5±1.5
